# Recent advances in understanding the epidemiology of healthcare-associated infections

**DOI:** 10.12688/f1000research.15891.1

**Published:** 2019-01-25

**Authors:** Pranavi Sreeramoju

**Affiliations:** 1 University of Texas Southwestern Medical Center at Dallas, Dallas, TX, USA

**Keywords:** healthcare-associated infections, infection prevention, updates in literature

## Abstract

Since the 2014 publication of updates to the Society for Healthcare Epidemiology of America (SHEA) compendium of strategies to reduce healthcare-associated infections, there have been several advances in understanding the epidemiology of these diseases. This review article captures many of the key advances but does not include all of them.

## Introduction

Nosocomial infections, more accurately referred to as healthcare-associated infections (HAIs), have gained increased attention from healthcare professionals as well as from patients and policy makers in recent decades. The transition in nomenclature away from the terms “nosocomial” or “hospital-onset” and toward “healthcare-associated” reflects increased identification of infections in healthcare settings outside hospitals, such as ambulatory surgical centers, dialysis centers, and nursing homes.

This article is a review of key advances in the epidemiology of HAI prevention since the publication of the updated Society for Healthcare Epidemiology of America (SHEA) compendium of strategies to reduce HAI ^1, 2^. In order to confine the scope, this article does not address HAIs associated with devices like external ventricular drains and left ventricular assist devices or recent hospital outbreaks such as *Mycobacterium chimaera* related to heater-cooler devices, *Candida auris*, and *Legionella*.

## Catheter-associated urinary tract infection

In a national collaborative program implemented in more than 10% of US hospitals to prevent catheter-associated urinary tract infection ^3^, infection rates in non-intensive care units fell from 2.28 to 1.54 infections per 1,000 catheter-days and catheter use decreased from 20.1% to 18.8%. This program used both technical interventions such as decreasing catheter use and cultural interventions using comprehensive unit-based safety program tools. In a multi-component initiative in 404 nursing homes, technical and socio-adaptive interventions were successful in reducing catheter-associated urinary tract infections by 54% and reducing urine culture orders by 15% ^4^.

## Surgical site infection

The Centers for Disease Control and Prevention (CDC) updated surgical site infection (SSI) prevention guidelines in 2017 ^5^. The key recommendations of the guidelines are the following. The revised antimicrobial prophylaxis recommendations, which had stewardship and risk versus benefit in mind, are more stringent. They clearly state that prophylaxis is indicated only for specific surgical procedures and that a bactericidal concentration of the antimicrobial agent(s) is important in the serum and tissues at the time of incision, including for cesarean section procedures. In previous years, antimicrobial prophylaxis for cesarean section was administered immediately after the umbilical cord was cut. An alcohol-based agent is the most effective agent for skin preparation in the operating room. The new guidelines recommend discontinuing antimicrobial prophylaxis after skin closure in the operating room for clean and clean-contaminated procedures, even in the presence of a drain. This new recommendation is different from the 24-hour window per previous guidelines. The guidelines also recommend against the application of topical antimicrobial agents to the surgical incision.

A chlorhexidine bath before surgery is a popular intervention. However, in a systematic review and meta-analysis of 243 primary studies ^6^, among which 8 were considered methodologically appropriate on the basis of the Jadad scale, chlorhexidine preoperative bathing was not associated with decreased risk of SSI. In this meta-analysis, a significant reduction in the infection rates was not found in a comparison study between patients subjected to preoperative bathing with 4% chlorhexidine versus placebo solution (relative risk 0.91, 95% confidence interval [CI] 0.76–1.09). The same absence of benefit was observed when chlorhexidine bathing was compared with soap (relative risk 1.06, 95% CI 0.68–1.66).

The importance of the different components of surgical attire in prevention of SSI is a subject of ongoing debate. In a thought-provoking article, Bartek *et al*. ^7^ firmly state that “there is no evidence regarding SSI risk related to operating room attire except for sterile gowns and the use of gloves” while humorously adding that naked personnel shed fewer bacteria. The importance of surgical technique was emphasized in a randomized, assessor-blinded trial on restrictive versus liberal fluid use during major abdominal surgery ^8^. The rate of SSI was 16.5% versus 13.6% ( *p* <0.0001) in the group with the use of 3.7 versus 6.1 L for intra-abdominal washout during surgery.

## *Clostridium difficile* infection

There have been several advances in the epidemiology of *Clostridium difficile* infection (CDI). Asymptomatic CDI is gaining a lot of attention. In a segmented time series analysis by Xiao *et al*. ^9^, isolating asymptomatic carriers in addition to isolating infected patients decreased the prevalence of isolation days for *C. difficile* from the pre-intervention period when surveillance for asymptomatic carriers was not performed. More data on the usefulness of probiotics have emerged. In an individual patient data meta-analysis with 6,851 participants from 18 placebo-controlled randomized clinical trials ^10^, probiotics reduced the odds of CDI by 0.35 (95% CI 0.23–0.55). Multi-species probiotics were more protective than single-species probiotics. During a period of piperacillin-tazobactam shortage, the incidence of hospital-onset CDI increased contrary to expectations because of a shift in usage to other high-risk antibiotics like carbapenems and higher-generation cephalosporins ^11^. In a study on the incidence of CDI during an initiative to accelerate and improve care for patients with sepsis, the incidence of CDI increased and this was controlled when a dedicated antimicrobial stewardship program was implemented ^12^. The hospital environment is a source of transmission of *C. difficile*. A secondary analysis of the results of the Benefits of Enhanced Terminal Room Disinfection study showed that the addition of ultraviolet light disinfection significantly reduced the risk of acquisition of *C. difficile* by 11% ** ^13^.

## Contact isolation

The SHEA published expert guidance on the duration of contact isolation for methicillin-resistant *Staphylococcus aureus* (MRSA), vancomycin-resistant *Enterococcus*, and extended-spectrum beta-lactamase-producing *Enterobacteriaceae* ^14^, recommending a shorter duration of contact isolation for most organisms except carbapenem-resistant *Enterobacteriaceae*. Several studies have shown a lack of increase in the incidence of multidrug-resistant organisms with shortening the duration of contact isolation ^15, 16^. These studies as well as a systematic analysis by Marra *et al*. ^17^ found that secular trends and the impact of horizontal measures outweighed the effect of contact precautions. Lin *et al*. ^18^ found that state-mandated active surveillance for MRSA did not reduce the prevalence of MRSA colonization.

## Reprocessing of endoscopes

No breaches in adherence to manufacturer guidelines for high-level disinfection of scopes were identified in an outbreak investigation of carbapenem-resistant *Klebsiella pneumoniae* with blaOxa-232 gene associated with endoscopic retrograde cholangiopancreatography (ERCP) in 17 patients ^19^. Reprocessing was less effective if the scope elevator mechanism was in a horizontal position as opposed to a vertical position during the high-level disinfection cycle in an automated endoscope reprocessor ^20^. In yet another study, intraluminal fluid was detected in 22 out of 45 endoscopes tested after the completion of high-level disinfection. Retained fluid with high adenosine triphosphate levels was found in 22% of endoscopes, and microbial growth was detected in 71% of endoscopes ^21^. In a study, remote video auditing with feedback using a 40-point checklist for getting ERCP reprocessing right was effective in ensuring that all steps were followed correctly ^22^. The challenge for generalizing the findings of this study would be a practical one, as the process of following a long checklist takes precious time and effort of personnel. We need more efficient ways of ensuring that high-level disinfection and sterilization yield expected levels of disinfection or sterilization.

## Antimicrobial resistance and stewardship

In a study that elucidated the epidemiology of carbapenem-non-susceptible *Acinetobacter baumannii* from a multi-city point prevalence survey within emerging infections program (EIP) sites, nearly half of the *Acinetobacter* strains isolated from persons with HAI reported to the CDC National Healthcare Safety Network in 2014 were carbapenem-non-susceptible ^23^. The study estimated that the incidence in the population surveyed was 1.2 per 100,000 patients during 2012 to 2015. Healthcare exposure within the previous year was present in 98% of cases, and an indwelling device, most often a urinary catheter, was present in 84% of cases; 17.9% of the patients died. The association between antimicrobial stewardship as a patient care improvement process, and improvement in patient outcomes as measured by hospital-onset multidrug-resistant bloodstream infections and *Candida* bloodstream infections, was shown in a study by Molina *et al*. ^24^.

## Preventing infection risk to healthcare personnel

In a study to assess the effectiveness of personal protective equipment (PPE) as a barrier to pathogen transmission, Kwon *et al*. ^25^ used fluorescence and MS2 bacteriophage to evaluate self-contamination while donning and doffing PPE. Overall, 27% of healthcare personnel (HCP) made at least one protocol deviation while donning and 100% while doffing PPE for Ebola virus disease (EVD). While using PPE for contact precautions, 50% and 67% of personnel, respectively, made protocol deviations while donning and doffing PPE. The study also identified protocol deviations by doffing assistants and trained observers.

In a multi-center study to evaluate the epidemiology of tuberculosis (TB) exposure in hospitals, 59.4% of patients were inadequately masked at the time of entry or inadequately isolated during hospital admission. These patients were more likely to be transplant recipients, have acid-fast bacilli on sputum stain, and have a chest radiograph with typical findings for TB and were less likely to have extrapulmonary TB ^26^. Although the concern for exposure to TB in healthcare settings is real, it does depend on the prevalence of TB disease seen in the healthcare facility. In a large medical center in the Midwest where 50 patients with TB disease received care in a 14-year period, only 0.3% of the 40,142 HCP who received a tuberculin skin test converted over 16.4 years, and no one developed TB disease ^27^. This study underscores the recommendation of the 2005 CDC guidelines for TB control ^28^ to determine the frequency of TB screening among personnel on the basis of incidence of TB in facilities.

## Healthcare personnel vaccination

In an outbreak investigation and control of mumps, a third dose of measles, mumps, and rubella (MMR) vaccine was effective in preventing mumps infection. The attack rate was 6.7 per 1,000 in those who got a third dose versus 14.5 per 1,000 in those who received two doses ( *p* <0.001) ^29, 30^. Although several institutions have already implemented mandatory influenza vaccination for HCP, effectiveness in reducing HCP absenteeism was published only in 2018. In a study conducted at outpatient settings in 3 university and 4 Veterans Affairs medical centers with 2,304 outpatient HCP at mandatory vaccination sites and 1,759 outpatient HCP at non-mandatory vaccination sites, vaccinated HCP had fewer sick days than non-vaccinated HCP (odds ratio 0.81, 95% CI 0.69–0.95) ^31^.

## Ebola transmission

Asymptomatic Ebola virus infection contributed very little to transmission on the basis of testing with an oral fluid anti-glycoprotein IgG assay with a specificity of 100% and a sensitivity of 95.9%. Of household contacts not diagnosed with EVD, 47.6% (229 out of 481) had high-level exposure (direct contact with a corpse, body fluids, or a case with diarrhea, vomiting, or bleeding). Among the household contacts, 11 out of 92 (12.0%, 95% CI 6.1–20.4) tested positive when contact occurred at the time the household member had EVD symptoms. By comparison, 10 out of 388 (2.6%, 95% CI 1.2–4.7) household contacts tested positive when contact occurred at the time the household member did not have symptoms ^32^. In another study, a HCP who was in flight when symptoms of EVD began did not transmit to 238 passengers on a flight from Sierra Leone to Glasgow with two stops ^33^. This is a little reassuring because of the high volume of modern-day air travel.

## Leadership in healthcare epidemiology

None of the healthcare epidemiology work is possible without leadership and competencies. Three articles from the SHEA address the necessary infrastructures, skills, and competencies that are helpful for someone to be an effective leader in healthcare epidemiology ^34– 36^. There is increased appreciation for synergies between infection prevention and antimicrobial stewardship ^37^. The Veterans Affairs system is building an implementation science infrastructure for infection prevention ^38^, and that is a step in the right direction. As we continue to push forward in the field, it is important to remember that we do not know all of the answers and that some answers may be unknowable. I will close this review with a reference to the challenges in managing patients who presented with suspected or confirmed Ebola virus infection at the National Institutes of Health ^39^; I think the approach applies to several other aspects of HAI prevention and control. “We answered questions saying, ‘We don’t know’, when we didn’t know the answer, but we promised to try to find the answer, if it existed. Alternatively, we noted mechanisms used to mitigate risks associated with our inability to answer a question with precision. The clinical leadership consistently offered a calm presence to staff who had anxieties. Institutions cannot ignore these anxieties, as they can become paralyzing”. Good leadership is necessary to reduce the burden of HAIs through implementation of known prevention approaches and to advance science and epidemiology in order to help further understand these infections.
